# Bergamottin a CYP3A inhibitor found in grapefruit juice inhibits prostate cancer cell growth by downregulating androgen receptor signaling and promoting G0/G1 cell cycle block and apoptosis

**DOI:** 10.1371/journal.pone.0257984

**Published:** 2021-09-27

**Authors:** Opalina Vetrichelvan, Priyatham Gorjala, Oscar Goodman, Ranjana Mitra

**Affiliations:** 1 Department of Biomedical Sciences, College of Medicine, Roseman University of Health Sciences, Las Vegas, Nevada, United States of America; 2 Comprehensive Cancer Centers of Nevada, Las Vegas, Nevada, United States of America; Universita degli Studi della Campania Luigi Vanvitelli, ITALY

## Abstract

Prostate cancer is the second leading cause of cancer related death in American men. Several therapies have been developed to treat advanced prostate cancer, but these therapies often have severe side effects. To improve the outcome with fewer side effects we focused on the furanocoumarin bergamottin, a natural product found in grapefruit juice and a potent CYP3A inhibitor. Our recent studies have shown that CYP3A5 inhibition can block androgen receptor (AR) signaling, critical for prostate cancer growth. We observed that bergamottin reduces prostate cancer (PC) cell growth by decreasing both total and nuclear AR (AR activation) reducing downstream AR signaling. Bergamottin’s role in reducing AR activation was confirmed by confocal microscopy studies and reduction in prostate specific antigen (PSA) levels, which is a marker for prostate cancer. Further studies revealed that bergamottin promotes cell cycle block and accumulates G0/G1 cells. The cell cycle block was accompanied with reduction in cyclin D, cyclin B, CDK4, P-cdc2 (Y15) and P-wee1 (S642). We also observed that bergamottin triggers apoptosis in prostate cancer cell lines as evident by TUNEL staining and PARP cleavage. Our data suggests that bergamottin may suppress prostate cancer growth, especially in African American (AA) patients carrying wild type CYP3A5 often presenting aggressive disease.

## Introduction

Prostate cancer (PC) is the second leading cause of cancer death in US men: according to the American Cancer Society, approximately 34,130 men will die of prostate cancer in 2021. Anti-cancer treatment often has adverse effects due to its action within normal tissues. Given the initial slow growing nature of prostate cancer and the front-line efficacy of androgen deprivation therapy (ADT), adjunctive therapy using dietary supplements with these ADT may provide us options to lower ADT dosage and reduce its adverse effects.

In this study we have tested the effects of bergamottin, a natural occurring furanocoumarin, found in grapefruit juice. Furanocoumarins are secondary metabolites produced in citrus fruits and they support a plant’s defense against pathogens [1–3]. The major furanocoumarins found in grapefruit are bergamottin, epoxybergamottin and 6’,7’ dihydroxybergamottin [4]. Furanocoumarins have been reported to have anticancer activity and are known to activate multiple signaling pathways leading to cell cycle arrest, apoptosis, and cell death [3, 5]. Bergamottin has been studied for its anti-proliferative effect on different cancer cell types as a dietary supplement [6–8].

Bergamottin, the furanocoumarin compound used in our current study, is also a strong inhibitor of CYP3A4/5, a P450 enzyme dominantly expressed in the liver [9–12]. Based on its crystal structure bergamottin shows a tendency towards π-π stacking which influences its interaction with the heme group of CYP3A4/5 [9]. CYP3A5 is the main extrahepatic P450 form expressed in both normal prostate and in prostate cancer [13]. Hence bergamottin, a CYP3A5 inhibitor, can play an important role in treating prostate cancer. The androgen receptor (AR) is the driving force of prostate cancer growth. In our previous studies we have shown that CYP3A5 expressed in intratumoral prostate regulates AR expression regulating prostate cancer cell growth [14]. We selected bergamottin for this study to monitor if it can be used as an CYP3A5 inhibitor to reduce AR activation and block prostate cancer growth.

Prostate cancer represents a major health disparity among African American (AA) men as new cases diagnosed are 60% higher in AAs as compared to Caucasian men (CA) [15, 16]. The expression of CYP3A5 is polymorphic, the most common mutant, CYP3A5 (*3), is expressed in 95% CA resulting in very low protein activity [17], AAs preferentially (74%) carry wild type (*1, form expressing full length active CYP3A5 protein) CYP3A5, which constitutively promotes AR activation potentially contributing to health disparity. CA men conversely carry the mutant form expressing very low levels of CYP3A5 protein.

As bergamottin is a CYP3A inhibitor its inhibitory effect can provide with a treatment strategy in AAs expressing high levels of CYP3A5 [18]. To test the effect of bergamottin on prostate cancer growth we used two cell lines one of AA origin (MDAPCa2b) expressing wild type CYP3A5 and the other of CA origin (LNCaP) carrying mutant CYP3A5. Bergamottin has been shown to inhibit cancer cell growth by regulating multiple pathways in different cancer types, stat3 pathway, PARP cleavage and apoptosis, PI3/Akt survival pathway, cyclinD1/cell cycle block and VEGF/angiogenesis inhibition [3, 19, 20]. So far it has not been tested for its ability to block AR activation which is very relevant in prostate cancer. In our recently published work we have shown how CYP3A5 inhibitors and inducers can alter AR activation more so in AAs expressing wild type CYP3A5 [21]. Understanding the biological mechanisms of how bergamottin regulates AR signaling in prostate cancer patients can help establish its mechanism based role in blocking prostate cancer growth especially in AAs expressing high CYP3A5 to reduce health disparity. In the current study we have examined if bergamottin can modulate the growth of the prostate cancer cells by altering AR signaling, prostate specific antigen (PSA) production, and cell cycle blockage.

## Materials and methods

### Cell lines, drugs and antibodies

LNCaP, MDAPCa2b and RWPE1 cells were purchased from ATCC and maintained in RPMI (Invitrogen, Carlsbad, CA), F-12K medium (ATCC® 30–2004) and Keratinocyte SFM media (Invitrogen, Carlsbad, CA) respectively. Supplements were added as recommended by ATCC. We have genotyped these two cell lines used in our current study. The LNCaP carries only mutant CYP3A5 (*3/*3) where as MDAPCa2b carries one wild type and one mutant CYP3A5 (*1/*3), producing higher levels of CYP3A5 full length protein [21]. The *3 splice variant results in a truncated protein due to the presence of a stop codon. The cell lines carrying the *3 variant can only produce some active protein (5–10% as compared to *1) by alternate splicing, bypassing the stop codon. Both the lines used in the study (LNCaP and MDAPCa2b) are androgen receptor positive and express PSA. Both LNCaP and MDAPCa2b cells are also androgen sensitive and show increased growth and higher PSA production with androgen induction. The LNCaP cells carry a mutation in the ligand binding site of AR receptor so it is able to bind promiscuously to a variety of steroids [22], where as MDAPCa2b cells are androgen independent [23].

Antibodies against Androgen receptor (ab74272, ab133273) were obtained from Abcam, (Cambridge, MA) anti-GAPDH (10R-G109A) was from Fitzgerald Industries (Acton, MA). Anti-α tubulin (2125S) and anti Lamin A/C (4C11), anti-PSA/KLK3 (D6B1) PARP (9542) and cell cycle regulation sampler kits (9932 and 9870) were obtained from Cell signaling technologies (Danvers, MA). The secondary antibodies (IR dye 680 and IR dye 800) were from LI-COR (Lincoln, NE). CYP3A inhibitor bergamottin (01338) was purchased from Sigma-Aldrich (St. Louis, MO).

### MTT assay

Growth of the cells was measured using a MTT assay. Cells were plated (2000 cells/ 96 well or 13,000 cells/ 24 well) for 48 hours then treated with bergamottin for 4 days (96 hours) and then incubated with MTT (3-(4,5-Dimethylthiazol- 2-yl)-2,5-Diphenyltetrazolium bromide) (4 mg/ml) for 2 h. at 37°C. DMSO (vehicle control for bergamottin) levels were compensated in each bergamottin treatment group. Cells were centrifuged at 2000 g for 10 min and the supernatant was discarded. The cell pellet was dissolved in 100/500 μl of DMSO depending on the well size. A plate reader was used to read the absorption at 540 nm. Experiments were performed in octuplet/quadruplet and repeated at least three times.

### Clonogenic assay

For clonogenic assay, cells were plated in 6 well plates (10,000 cells) for 48 h and then treated with indicated amounts of bergamottin and vehicle control (DMSO). After 3 weeks, the colonies were fixed, stained (0.75% crystal violet, 50% ethanol and 1.75% formaldehyde) and counted. The assay was repeated three times.

### Western blotting

Cells were plated at 25–30% confluence when maintained in complete media. When the cells required DHT tretment and media replacement to charcoal stripped serum media (CSSM) they were plated at 35–40% confluence. Bergamottin is dissolved in DMSO (vehicle control) and the amount of DMSO at each treatment group was kept constant. After the completion of the treatment cells were washed in phosphate buffer (PBS) containing 2mMEDTA and 2mM EGTA and lysed in RIPA buffer (50 mM TRIS, 150 mM NaCl, 1% NP40, 0.5% sodium deoxycholate, 0.1% SDS, 2mM EDTA, 2mM EGTA) supplemented with protease and phosphatase inhibitors for total protein isolation. Protein estimation was performed using micro BCA assay and equal protein amponts were loaded on SDS-PAGE gels for western analysis. GAPDH was used as an internal control for total protein. Infrared fluorescent-labeled secondary antibodies were used for detection using Odyssey CLx. The bands were quantified using Odyssey software, which calculates pixel density and automatically takes an area adjacent to each band for background corrections.

### Cell fractionation

Nuclear and cytoplasmic cell fractionation was prepared using NE-PER Nuclear and cytoplasmic extraction kit from Thermo Scientific (Cat no.78833) and manufacturer’s instructions were followed. When the cells were treated with DHT, the media was changed to charcoal stripped serum media without phenol red (CSSM) 48 hours prior to the treatment. Bergamottin and DHT were added at specified concentration and duration as indicated. Cells were washed in PBS once before cells were suspended in CER buffer. Protease, phosphatase inhibitors and EDTA was added prior to cell lysis. The pellet remaining after cytoplasmic isolation was washed twice with PBS. The pellet was suspended in NER buffer for nuclear fraction extraction according to guidelines, samples were stored at -80°C until further processing. Tubulin and Lamin were used as internal controls for cytoplasmic and nuclear fractions respectively.

### Confocal microscopy

Cells were seeded into 35 mm Glass bottom dish (Cellvis catalog# D35C4-20-1.5-N) in complete media; media was changed to CSSM prior to DHT treatment. The cells were fixed in 4% paraformaldehyde for 20 minutes and permeabilized using permeabilizing buffer (0.2% Tween 20 in PBS) for 5 minutes. Cells were blocked using 10% goat serum diluted in permeabilizing buffer with 1% BSA for 15 minutes. Primary antibodies were diluted at 1:100 in staining buffer (1% BSA in PBS) and incubated for 2 hours at room temperature. Cells were washed three times (5 minutes each) in PBS. Secondary antibodies, TRITC-conjugated Donkey Anti-rabbit (711-025-152) was from Jackson immuno research, West Grove, PA was diluted at 1:100 in staining buffer and incubated for 60 minutes at room temperature. Cells were washed three times (5 minutes) in PBS and stained with 1 μg/mL DAPI (4’, 6-diamidino-2-phenylindole) in PBS for 5 minutes at room temperature. The cells were stored in PBS at 4°C until imaging was completed.

Cells were imaged using confocal laser scanning microscopy on a Nikon A1R using a galvano scanner and a 60× Apo-TIRF oil immersion objective. NIS Elements software form Nikon was used for recording the data.

### SiRNA transfection

Cells were plated in complete media without antibiotics on poly D-lysine-coated plates (80,000 cells per 6 well). After 48 hrs. of plating cells were transfected with smart pool non-target (NT) control siRNA (Dharmacon catalog# D-001810-10) and a pool of four siRNA (Dharmacon catalog# L-009684-01) against the CYP3A5using RNAimax, manufacturer’s instructions were followed. The final concentration of the siRNA (NT and target) used was 30 nM. The cells were processed for cell cycle and western analysis 96 hours after the transfection.

### Cell cycle assay and analysis

Cell cycle analysis was performed after staining the cells with propidium iodide (PI). For long term bergamottin treatment (96 hrs.) cells were plated at 25–30% confluence and for short term bergamottin treatment (48 hrs.) they were plated at 30–35% confluence. After completion of specified treatment the cells were trypsinized washed with PBS and fixed in 70% ethanol and then stained with PI in nicoletti buffer (propidium iodide 50 μg/ml, 0.1% sodium citrate, 0.1% triton X-100, RNase 1 mg/ml, in DPBS). Cells were analyzed using C6 Accuri flow cytometer (Becton Dickinson, Mountainview, CA). FlowJo software was used to gate and remove the debris (gate on SSC and FSC) and aggregates (FL2 A and FL2H) from the analysis. Watson pragmatic and and Dean-Jett -Fox methods was used to assign percentage frequencies to cells present in the different cell cycle stages.

### Apoptosis detection assay

To detect apoptosis in the cells after bergamottin treatment In Situ cell death detection kit from Sigma-Aldrich (St. Louis, MO) was used. This apoptosis detection kit fluorescently labels the single and double-stranded DNA breaks caused due to apoptosis using terminal deoxynucleotidyl transferase (TUNEL-reaction). The cells were plated in poly- D- lysine coated 35 mm Glass bottom dish (Cellvis catalog# D35C4-20-1.5-N), treated with bergamottin for the specified time, and dosage and then stained with the kit reagents. The kit recommended protocol was followed. After the staining, the confocal microscope was used to procure images of the cells. The TUNEL assay preferentially labels apoptosis in comparison to necrosis and positive apoptosis is recognized when cells incorporate fluorescent labelled nucleotide (Ex 450 to 500nm and Em 515 to 565-green).

## Results

### Bergamottin blocks growth of prostate cancer cell lines

**Fig 1:**
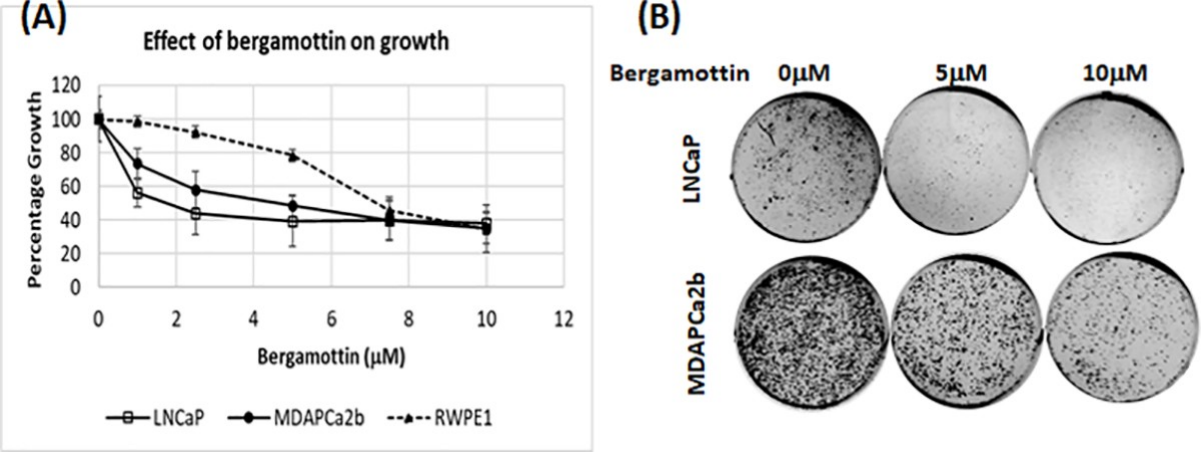
Bergamottin blocks prostate cancer cell growth. (A) Cells were treated with bergamottin or vehicle control (DMSO) for 120 hours; 48 hours after plating. MTT assay was performed as described in methods at the end of the treatment, the OD540 values were converted to relative percentage growth and plotted. (B) Clonogenic assay showing number of colonies formed after 3 weeks of bergamottin and vehicle treatment in both the cell lines. The colonies were fixed and stained with crystal violet for visualization. Both the experiments were repeated three times and data from a representative experiment is shown.

We tested the effects of bergamottin on prostate cancer cells using MTT and clonogenic assays. Since bergamottin is, a known CYP3A inhibitor we used two separate prostate cancer cell lines to test its effect differentially: LNCaP, of Caucasian origin carrying mutant CYP3A5 and expressing low CYP3A5, and MDAPCa2b, of African American origin and carrying one wild type CYP3A5 and expressing higher levels of CYP3A5 active protein. MTT assay revealed that bergamottin reduced prostate cancer cell growth. The 50% growth reduction was observed in LNCaP and MDAPCa2b cells with 2.4 μM and 4 μM bergamottin treatment respectively (Fig 1A). Growth assay also revealed that bergamottin does not effect growth of RWPE1 cells (non transformed prostate epithelium) at lower concentration although at higher concentration (10μM) all the three cell lines show 60% inhibition of growth. The growth inhibition effect of bergamottin was further confirmed using long term clonogenic assays. Bergamottin significantly reduced the number of colonies in both the cell lines, LNCaP and MDAPCa2b, at both 5μM and 10μM concentrations (Fig 1B).

### Bergamottin reduces androgen expression, AR activation and PSA production

**Fig 2:**
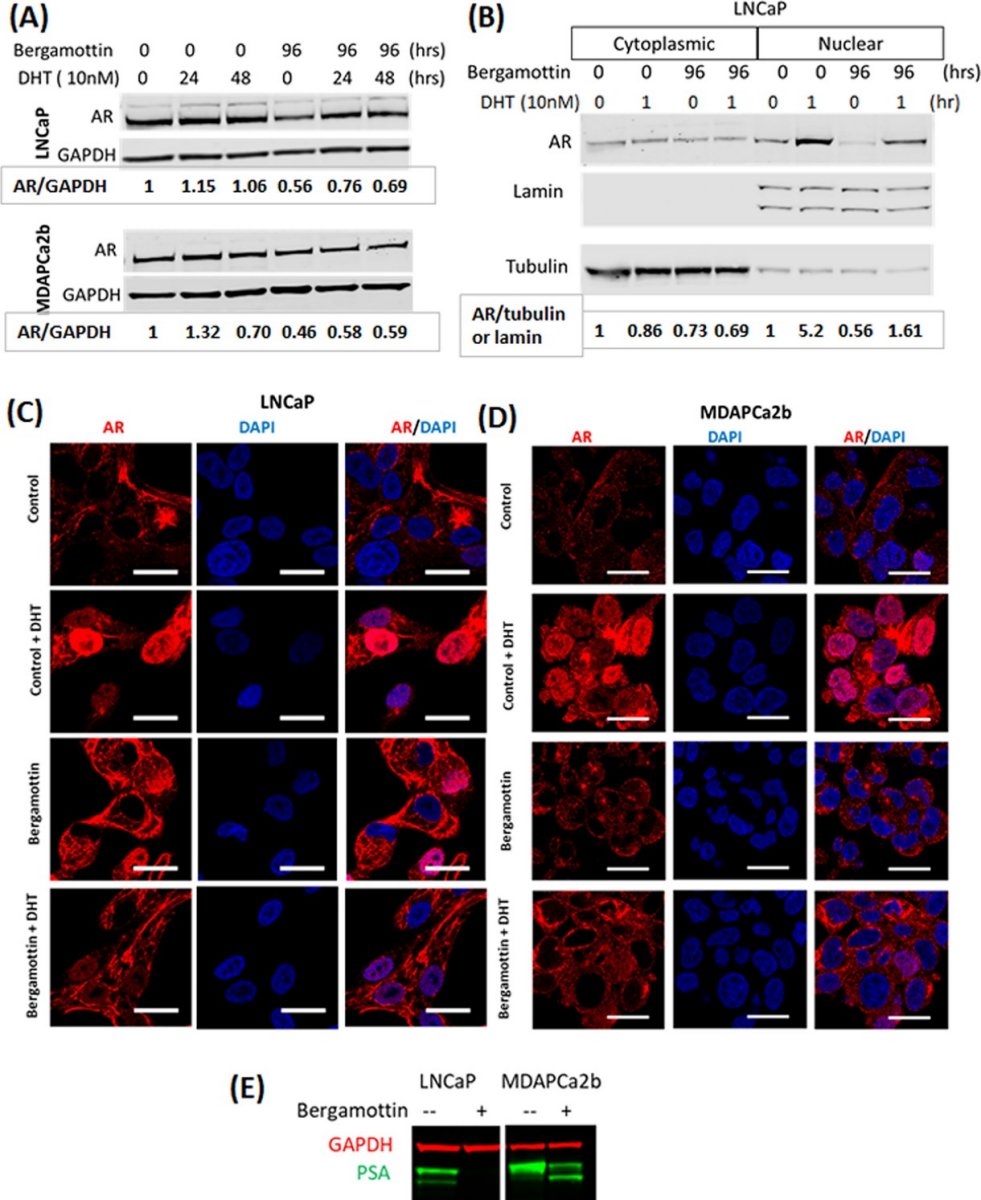
Bergamottin downregulated total AR expression, AR activation and downstream signaling: (A) Western blotting showed that bergamottin reduces total AR. GAPDH was used as an internal control for equal loading. Cells were plated in complete media, replaced with charcoal stripped serum and phenol red free media (CSSM) 24 hours after plating. Bergamottin (10μM) and vehicle control (DMSO) was added at the time of replacing with CSSM. DHT was added either 48 or 72 hours after replacing with CSSM depending on the treatment duration. (B) Analysis of AR nuclear localization shows that bergamottin reduces AR in nuclear fraction with or without DHT induction. Lamin and tubulin have been used as internal controls. The plating and bergamottin treatment time point was similar as (A) the DHT tretment was given 95 hours after replacement with CSSM. (C&D) Confocal microscopy showing effect of bergamottin on AR nuclear localization. Cell plating and treatment was similar to (B). Size bar 20μm. (E) Western blotting showing reduction in PSA production with bergamottin treatment (10μM, 96 hours).

Androgen receptor is the driving force of prostate cancer growth. Since we observed that bergamottin blocked prostate cancer cell growth, we tested its effect on AR expression and signaling. Bergamottin downregulated total AR levels in both LNCaP and MDAPCa2b cells by 50% (Fig 2A). DHT slightly increased the total AR expression in DMSO and bergamottin treated LNCaP and MDAPCa2b cells. Cell fractionation experiments with LNCaP cells showed that bergamottin inhibited nuclear translocation of AR consistent with CYP3A5 siRNA inhibition reported earlier: nuclear AR with and without DHT induction was lower in the bergamottin treated cells compared to the control set (Fig 2B). The inhibition of nuclear translocation of AR was further confirmed in both the cell lines using confocal microscopy studies (Fig 2C and 2D). In bergamottin treated cells, active AR migration to the nucleus with DHT induction was significantly reduced versus the control (DMSO) treated cells. Interstingly, we observed this pattern of increased cytoplasmic AR in our confocal images with bergamottin similar to other inhibitor treatments (e.g. ritonavir, [21]) observed earlier. Bergamottin also significantly reduced PSA levels in both cell lines which is a readout for AR downstream signaling as PSA expression is regulated by AREs (androgen response elements) (Fig 2E).

### Bergamottin induces cell cycle arrest

**Fig 3:**
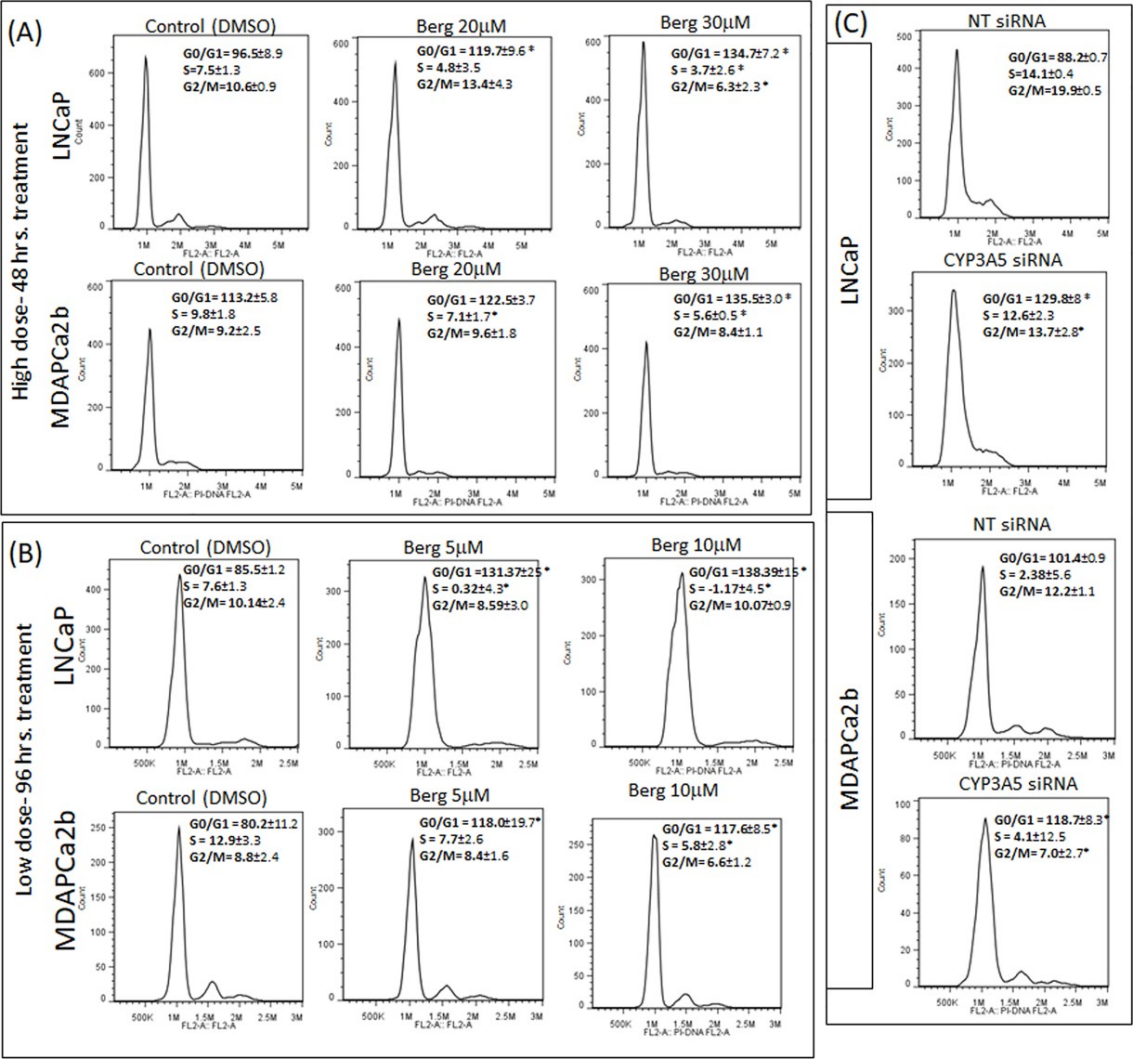
Bergamottin treatment causes accumulation of G0/G1 cells similar to CYP3A5 siRNA treated cells. Watson Pragmatic cell cycle analysis showing frequency of G0/G1, S, and G2/M population in the vehicle control (DMSO), bergamottin and siRNA treated cells. (A and B) Cells were treated with indicated concentration of bergamottin for indicated time points. The cell cycle analysis was performed as described in methods and the analysis was done using FlowJo software, a representative experiment is shown with Watson pragmatic analysis. The average frequency of each population and SD values (±) were calculated after analysing the replicates (minimum of three) and is indicated next to each treatment conditions. (*)- indicates that the P values was less than 0.05 when compare with the respective controls. (C)- Cells were treated with either non target (NT) pool siRNA or CYP3A5 siRNA pool. The analysis method is same as in A and B.

Since bergamottin reduces cell growth we tested its effect on the cell cycle. The cells were treated with either higher concentration (20μM and 30μM) of bergamottin for a shorter duration (48 hours) (Fig 3A) or lower concentration (5μM and 10μM) of bergamottin for longer duration (96 hours) (Fig 3B). It has been shown earlier that the IC_50_ for Bergamottin CYP3A5 inhibition is 20μM [11], to compensate for the shorter time (48 hrs.) of treatment we have increased the dosage in line with the Ki value. Cell cycle analysis revealed accumulation of cells in the G0/G1 phase in both high and low dose treatment groups in both the cell lines tested (Fig 3A and 3B, Table 1). With 30μM (48 hrs.) and 10μM (96 hrs.) bergamottin treatment we also observed significant reduction of S-phase cells as compared to the control (Fig 3A and 3B). Both the analysis methods Watson pragmatic and Dean-Jett-Fox show accumulation of cells in the G0/G1 phase and reduction in S phase cells in both LNCaP and MDAPCa2b cells with one exception (LNCaP 96 hrs treatment Dean-Jett-Fox analysis). Inhibition of CYP3A5 using an CYP3A5 siRNA pool also showed same accumulation of G0/G1 polulation and depletion of S phase cells in both the cell lines (Fig 3C and Table 1) with both analyses. Both bergamottin treatment and CYP3A5 siRNA treatment show a similar accumulation of G0/G1 population and depletion of S phase cells, consistent with inhibition of CYP3A5 by bergamottin.

**Table 1:** Cell cycle analysis showing effect of bergamottin and CYP3A5 siRNA treatment in LNCaP and MDAPCa2b cells.

**LNCaP (48 hrs. bergamottin treatment)**														
		**Dean-Jett-Fox**					**Watson Pragmatic**							
		**G1**		**S**	**G2**		**G1**			**S**				**G2**
**DMSO**		75.7 ±3.2		9.7 ±4.8	11.8 ±0.7	**DMSO**	96.5 ±8.9			7.5 ±1.3				10.6 ±0.9
**Berg (20μM)**		**86.7** ±2.3		-**9.8** ±3.0	**15.3** ±1.0	**Berg (20μM)**	**119.7** ±4.6			4.8 ±3.5				13.4 ±4.3
**Berg (30μM)**		**96.2** ±3.6		**-8.4** ±4.5	**7.7** ±0.9	**Berg (30μM)**	**134**.**7** ±3.2			3.7 ±2.6				**6.3** ±2.3
**LNCaP (96 hrs. bergamottin treatment)**														
	**Dean-Jett-Fox**						**Watson Pragmatic**							
	**G1**		**S**		**G2**		**G1**			**S**			**G2**	
**DMSO**	83.7±0.8		1.23 ±0.8		14.5 ±0.6	**DMSO**	85.51 ±1.2			7.6 ±1.3			10.14 ±2.4	
**Berg (5μM)**	**90.81** ±1.7		-0.3 ±2.3		**10.53** ±1.0	**Berg (5μM)**	**131.37** ±25			**0.32**±4.3			8.59 ±3.0	
**Berg (10μM)**	**95.1** ±3.4		-0.03 ±1.1		**0.22** ±0.4	**Berg (10μM)**	**138.39** ±15			**-1.17** ±4.5			10.07 ±0.9	
**MDAPCa2b (48 hrs. bergamottin treatment)**														
		**Dean-Jett-Fox**							**Watson Pragmatic**					
		**G1**		**S**	**G2**				**G1**			**S**		**G2**
**DMSO**		76.8 ±1.9		11.4 ±1.7	10.5 ±1.3	**DMSO**			113.2 ±5.8			9.8 ±1.8		9.2 ±2.5
**Berg (20μM)**		**84.9** ±0.4		**6.7** ±0.7	9.6 ±0.5	**Berg (20μM)**			122.5 ±3.7			**7.1** ±1.7		9.6 ±1.8
**Berg (30μM)**		**88.4** ±3.6		**3.3** ±3.4	6.8 ±3.2	**Berg (30μM)**			**135.5** ±3.0			**5.6** ±0.5		8.4 ±1.1
**MDAPCa2b (96 hrs. bergamottin treatment)**														
		**Dean-Jett-Fox**						**Watson Pragmatic**						
		**G1**		**S**	**G2**			**G1**			**S**			**G2**
**DMSO**		81.3 ±1.9		12.7 ±1.7	3.6 ±2.6	**DMSO**		80.2 ±11.2			12.9 ±3.3			8.8 ±2.4
**Berg (5μM)**		**84.2** ±1.5		12.2 ±1.0	2.3 ±2.2	**Berg (5μM)**		**118.0** ±19.7			7.7 ±2.6			8.4 ±1.6
**Berg (10μM)**		**88.8** ±2.0		**7.4** ±2.5	3.3 ±0.3	**Berg (10μM)**		**117.6** ±8.5			**5.8** ±2.8			6.6 ±1.2
**LNCaP (siRNA treatment)**														
		**Dean-Jett-Fox**					**Watson Pragmatic**							
		**G0/G1**		**S**	**G2/M**		**G0/G1**			**S**				**G2/M**
**NT siRNA**		66.7±0.6		15.2±3.2	18.3±3.1	**NT siRNA**	88.2±0.7			14.1±0.4				19.9±0.5
**CYP3A5 siRNA**		**81.6**±0.8		12.4±2.5	**2.8**±3.2	**CYP3A5 siRNA**	**129.8**±8			12.6±2.3				**13.7**±2.8
**MDAPCa2b (siRNA treatment)**														
		**Dean-Jett-Fox**					**Watson Pragmatic**							
		**G0/G1**		**S**	**G2/M**		**G0/G1**			**S**				**G2/M**
**NT siRNA**		75.8±1.5		11.3±6	9±8.5	**NT siRNA**	101.4±0.9			2.3±5.6				12.2±1.1
**CYP3A5 siRNA**		**89.9**±6.7		8.3±4.5	2.0±1.9	**CYP3A5 siRNA**	**118.7**±8.3			4.1±12.9				**7**±2.7

Table showing increase in G0/G1 population and decrease in S phase cells with bergamottin and CYP3A5 siRNA treatment in both cell lines using both cell cycle analysis methods: Dean-Jett-Fox and Watson pragmatic. The values represent average of minimum of three independent experiments, ± indicates SD values. The values written in bold indicate P value of less than 0.05 when compared with respective controls.

### Western blot analysis showing differential expression of cell cycle regulating proteins after bergamottin treatment

**Fig 4:**
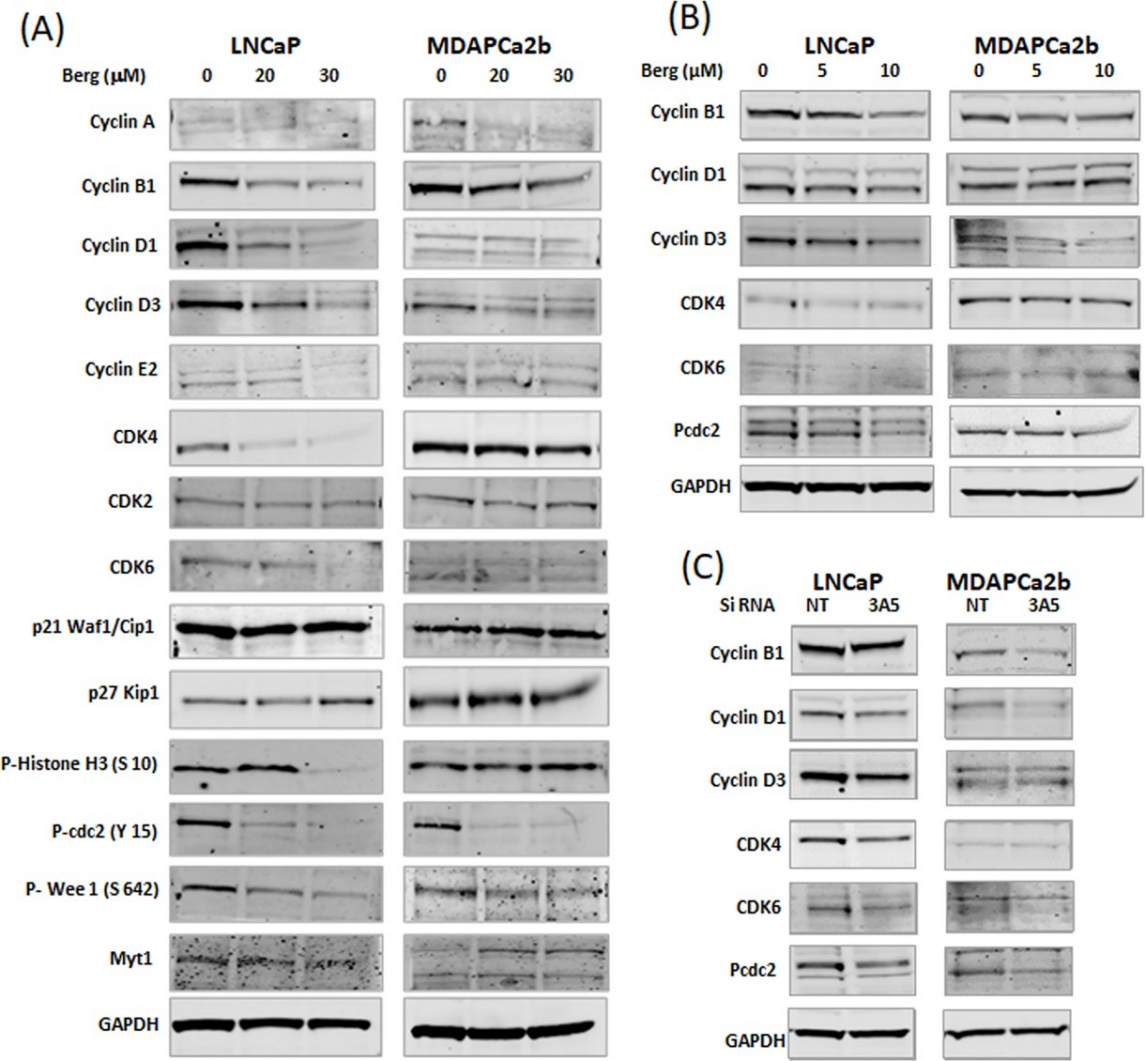
Differential regulation of cell cycle regulating protein after bergamottin treatment. (A &B) Western blot analysis of control and bergamottin treated cells depicting changes in cell cycle regulating proteins. Treatment was either given for 48 hrs. (high dose, 20 and 30μM) or 96 hrs. (low dose, 5 and 10μM) as described in Fig 3A and 3B. (C) the effect of CYP3A5 siRNA on cell cycle regulating proteins as compared to NT (non-target) control siRNA pool treatment (96 hours). GAPDH has been used as a loading control. The experiment was repeated three times, a representative replicate is shown.

Cell cycle arrest occurs due to block at one of the four check points: G1/S check point, G2/M check point, SAC check point, intra S check point and restriction point (reversible). All these check points are regulated by cyclins and CDKs, we tested the effect of bergamottin on these cell cycle regulating proteins. We first tested effects of bergamottin (20 and 30 μM) on cyclins and CDKs and then on proteins regulating cyclins and CDKs. Our western blot analysis revealed reduction of cyclins B1, D1, D3 and E in LNCaP cells and reduction of cyclins A, B1 and D3 in MDAPCa2b cells (Fig 4A, Table 2). We observed reduction of CDK4 and CDK6 in LNCaP and only CDK4 in MDAPCa2b cells. The fold changes after GAPDH calibration are shown in Table 2. The effect on the cyclin B1, D and CDK4 and CDK6 were tested with low concentration bergamottin treatment (5 and 10 μM) as they were altered with higher doses of bergamottin. Our results indicate that similar to high dose treatment, cyclin B1 and D3 levels were lower with low dose bergamottin treatment in both lines and cyclin D1 were lower only in LNCaP cells (Fig 4B). We did not observe any significant change in CDK4 or CDK6 levels with low dose of bergamottin in both the cell lines. We also tested the effect of CYP3A5 inhibition using siRNA on cyclins and CDKs regulating cell cycle, we observed that cyclin B, D1 and D3 were down regulated in LNCaP similar to bergamottin treatment (Fig 4C). In MDAPCa2b cells we observed down regulation of cyclin B1 and D1 which was slightly different than the pattern observed with bergamottin treatment (cyclin B1 and D3). Both CDK4 and 6 were downregulated in LNCaP cells where as only CDK6 was down regulated in MDAPCa2b cells with CYP3A5 siRNA treatment compared to NT control.

**Table 2:** Fold changes in cell cycle regulating proteins after bergamottin treatment.

	LNCAP			MDAPCa2b		
	Bergamottin (μM)			Bergamottin (μM)		
	**0**	**20**	**30**	**0**	**20**	**30**
**Cyclin A**	100	80	55	100	86	58
**Cyclin B1**	100	50	44	100	52	42
**Cyclin D1**	100	43	12	100	106	110
**Cyclin D3**	100	68	44	100	55	49
**Cyclin E**	100	109	53	100	120	87
**CDK2**	100	62	67	100	63	77
**CDK4**	100	46	30	100	90	81
**CDK6**	100	60	12	100	108	98
**p21**	100	83	102	100	139	126
**p27**	100	112	183	100	158	143
**P-histone H3**	100	294	35	100	130	152
**P-cdc2**	100	18	5	100	14	15
**P-Wee1**	100	63	37	100	80	87
**Myt 1**	100	103	82	100	259	451

Table represents normalized fold changes after GAPDH calibration and corresponds to the replicate shown in Fig 4A (high dose and 48 hrs. treatment).

Additionally, we observed increase in p27kip1 expression in both cell lines and no changes were observed in p21 levels. Decreased phosphorylation of cdc-2 (Y15) and wee-1 (S642) were also observed in both cell lines (Fig 4A). Decreased cdc-2 phosphorylation was observed with low dose bergamottin treatment and also with CYP3A5siRNA inhibition in both the cell lines (Fig 4B). Changes in phosphorylation of Histone H3 (S10) and Myt1 levels differed between the two cell lines with bergamottin treatment.

### Bergamottin causes cell death by triggering apoptosis in the cells

**Fig 5:**
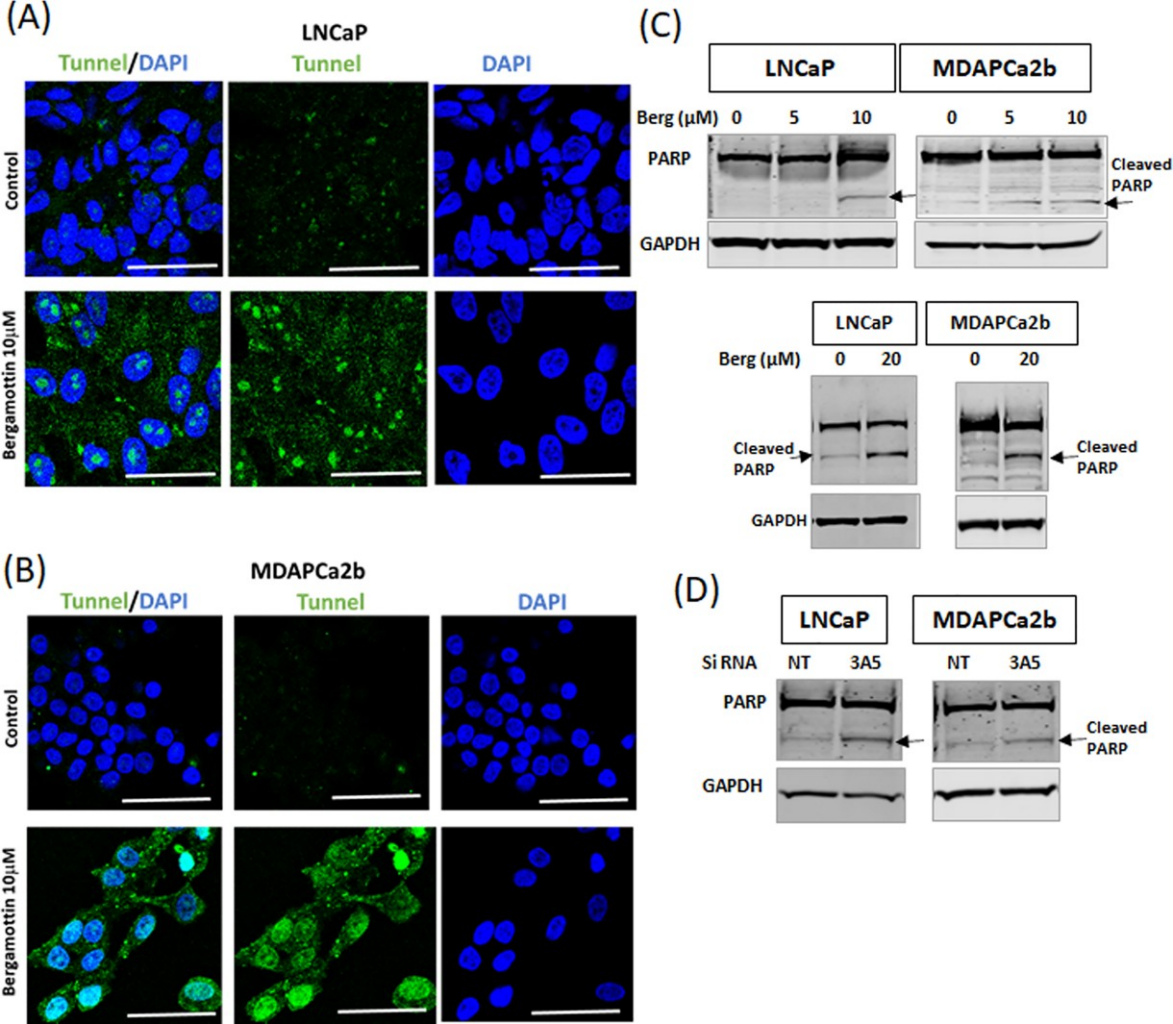
Bergamottin causes apoptosis in LNCaP and MDAPCa2b cells. (A & B) LNCaP and MDAPCa2b cells were treated with bergamottin (10μM) or DMSO (control) for 4 days. Cells were then fixed and stained for tunnel assay reagents as described in methods. Experiment was repeated three times and a representative experiment is shown. Size bar 50μm. DAPI was used to stain the nucleus. (C) Western blot analysis showing cleaved PARP after bergamottin treatment (5 and 10 μM for 96 hrs. and 20 μM for 48 hrs.), GAPDH was used as an internal control. (D) Western blot showing PARP cleavage after CYP3A5 siRNA and NT (non-target) siRNA treatment (96 hrs.).

Since we observed cell cycle blockade and growth inhibition with bergamottin treatment, we wanted to test if it is triggering apoptosis. To assess apoptosis we performed tunnel assays with both the cell lines (Fig 5A & 5B). The tunnel assay indicates DNA breaks (green fluorescence) due to apoptosis with bergamottin treatment in both cell lines. Both the cell lines show increased DNA breaks with bergamottin treatment, although the pattern of tunnel staining was different between the two lines. In LNCaP we observed punctate nuclear staining, whereas in MDAPCa2b we observed a diffuse staining pattern. We next tested the effect of bergamottin treatment on PARP cleavage (Fig 5C). Both low dose and high dose bergamottin were tested for PARP cleavage and showed apoptosis (cleaved PARP) although it was more pronounced at the higher dosage. Similar PARP cleavage was observed in CYP3A5 siRNA treated LNCaP and MDAPCa2b cells indicating that CYP3A5 inhibition triggers apoptosis (Fig 5D).

## Discussion

Our results indicates that bergamottin inhibits growth in both LNCaP and MDAPCa2b cells at significantly low concentrations (50% inhibition, 2.4 and 4μM respectively). Previously we have used CYP3A5 siRNA treatment to show CYP3A5s effect on growth using viable cell count, MTT, and clonogenic assays [14, 21]. Bergamottin has been shown to reduce growth of several other cancer cell types but the IC_50_ were reported in the range of 30–100μM [19, 20, 24–32]. The clonogenic assays also show significant loss of growth at 5 and 10 μM concentrations. This may be because both the cells express CYP3A5 active protein and bergamottin is a potent CYP3A5 inhibitor. We do observe that bergamottin inhibits LNCaP cells at lower concentrations as compared to MDAPCa2b. This may be attributed to lower levels of CYP3A5 protein expressed in LNCaP due to *3/*3 mutation present as compared to MDAPCa2b which carries one copy of wild type of CYP3A5 (*1/*3) [21]. Compared to LNCaP, MDAPCa2b may need higher concentration of bergamottin, due to expression of higher quantity of active full length CYP3A5 protein, to cause comparable growth inhibition. The other major difference between the previously reported results in the DU145 AR negative prostate cancer cell line [19] suggests bergamottin’s action is CYP3A5—AR signaling dependent [14]. Bergamottin does not effect RWPE1 (non-transformed prostate epithelium) cells at lower concentrations as much as it does the other two cancer cell lines which is consistent with the earlier reported data [19].

Although primarily bergamottin is a CYP3A4 inhibitor it also inhibits CYP3A5, CYP2B6 and CYP1A1 [9, 11, 12, 33, 34]. Expression of P450 CYPs is tissue specific and CYP3A5 is the major isoform expressed in both normal and prostate tumor [13]. Since CYP3A5 is the major P450 CYP isoform expressed in the prostate tissue, the effects observed in our experiments is primarily due to inhibition of CYP3A5 in the tested prostate cancer cell lines [13, 35]. The other major extrahepatic P450 isoform expressed in normal and prostate tumor is CYP1A1 [36], bergamottin is a competitive inhibitor of CYP1A1 and can aid in cancer chemoprevension due to CYP1A1s role in carcinogenesis, this inhbitory effect is independent of bergamottins affect on CYP3A5 and AR [33]. CYP3A4 and CYP2B6 are also inhibited by bergamottin but are not expressed in normal and prostate tumor cells.

Previously we have shown that CYP3A5 siRNA pool treatment blocks AR nuclear translocation [14] and had no response to growth with DHT induction which is dependent on AR nuclear translocation. Bergamottin which is a CYP3A inhibitor [6, 10, 37] also shows reduced nuclear AR consistent with our earlier results. In addition, we have also shown how the cell growth is inhibited by azamulin, a CYP3A4/5 inhibitor similar to bergamottin. Although AR expression is not increasing in the nucleus our confocal studies show increased cytoplasmic AR with bergamottin treatment compared to control. We have previously observed similar increase in cytoplasmic AR with certain inhibitor treatments (ritonavir) [21]. We believe that AR is getting trapped in the tubulin channels and not getting recycled normally, additional studies e.g. tubulin colocalization will be needed to fully understand this observation. In addition to reduced nuclear AR, bergamottin also lowers total AR expression which may be further contributing to growth inhibition, as AR is known to be the driver of prostate cancer growth. We observed loss of PSA in the bergamottin treated cells, with PSA levels representing a downstream readout of AR signaling [38]. In our recent publication we have demonstrated that CYP3A5 inducers promote AR nuclear translocation and PSA expression where as CYP3A5 inhibitors reduce AR nuclear migration and PSA expression [21]. Simultaneous treatment with CYP3A5 siRNA pool and CYP3A5 inducer nullified increased AR translocation and PSA when compared to CYP3A5 inducer treatment alone showing that the increased AR nuclear translocation and PSA production are specific to changes in CYP3A5. PSA expression is AR dependent since *Psa* carries AREs (androgen response elements) upstream and responds to androgen induction. This observation can be useful in patients with high AR and CYP3A5 expression such as African Americans, who preferentially carry wild type CYP3A5 and express higher AR, accompanied by relatively aggressive disease.

Our experiments show accumulation of cells in the G0/G1 phase (low and high dose of bergamottin) and depletion of S phase cells with both high and low dose bergamottin treatment. We do observe that the effect on cell cycle is more pronounced in LNCaP cells as compared to MDAPCa2b cells, this may be due to presence of high levels of CYP3A5 in MDAPCa2b cells that may require higher dosage of bergamottin for inhibition. Similar accumulation of G0/G1 cells were observed with CYP3A5 siRNA pool treatment as compared to NT pool (Fig 3C). Since bergamottin is a CYP3A5 inhibitor; simultaneous inhibition with CYP3A5 siRNA pool and bergamottin treatment cannot decipher if the cell cycle block is due to bergamottin’s effect on AR nuclear translocation or is independent.

Accumulation of G0/G1 cells can occur at the restriction point or at the G1/S check point [39]. The restriction point is regulated by cyclin D and CDK4,6 [40]. Since we observed reduction in Cyclin D1, D3 and CDK 4,6 in LNCaP cells and Cyclin D3 and CDK4 in MDAPCa2b cells (Fig 4A, Table 2) it shows that the block is at the restriction point in both the cell lines. The decreased expression of the cyclins is consistent in both the lines even with the lower dose of bergamottin treatment (Fig 4B). Reduction in Cyclin B1, D1 D3 in LNCaP and cyclin B1, D1 in MDAPCa2b and reduction in CDK4 levels (both lines) were observed with CYP3A5 siRNA treatment resulting in G0/G1 accumulation similar to bergamottin treatment. Additionally, we observed increase in p27kip1 which indicates increased population of quiescent cells (G0). Cyclin E and CDK2 are known to regulate G1/S check point which also leads to accumulation of G0/G1 cells, which is consistent with larger block observed in LNCaP cells which show downregulation of cyclin E2. Additionally, in LNCaP both Cyclin D1 and D3 are down regulated and in MDAPCa2b only Cyclin D3 is, which explains more pronounced accumulation of G1 cells in LNCaP as compared to MDAPCa2b. We also observed slight reduction in Cyclin A in bergamottin treated MDAPCa2b cells, cyclin A levels increase in S phase and is associated with intra S check point which can explain the why the loss of S phase cells is not evident in MDAPCa2b cells as compared to LNCaP. Both the cell lines showed reduced expression of cyclin B after bergamottin treatment which is associated with G2/M check point. Although we observe changes in the cyclin B but our cell cycle analysis does not show any increase in G2/M cell population. We observed decreased phosphorylation of cdc-2 (Y15) in bergamottin treated cells, phosphorylation at Y15 is known to block mitotic entry. It is possible that the reduction in cyclin B and cdc-2 dephosphorylation cancel each other out and we do not see any accumulation of G2/M cells [41]. We observed less phosphorylation of wee-1 (S642) which is a Y15 kinase consistent with the loss of phosphorylation of cdc-2. There is loss of phosphorylation of Histone H (S10) in the 30uM bergamottin treated LNCaP cells which is essential for chromosomal condensation for segregation during mitosis. Bergamottin has been shown to increase sub-G1 population in myeloma cells and cause G2/M block in lung cancer cells at higher concentrations [19, 20].

Bergamottin induces apoptosis in both prostate cancer cell lines, evidenced by tunnel assay staining of late apoptotic cells by labeling DNA breaks and nicks by Terminal deoxynucleotidyl transferase, which incorporates fluorescein UTP at 3’-OH ends in a template independent manner. Tunnel assay preferentially labels DNA strand breaks generated during apoptosis which allows discrimination of apoptosis from necrosis. Both the cell lines show apoptosis after bergamottin treatment, although MDAPCa2b cells have more fluorescein UTP incorporation than in LNCaP cells. Due to the discrepency of tunnel staining between both the cell lines PARP cleavage was investigated. We observed PARP- cleavage, an hallmark of apoptosis, with both bergamottin and CYP3A5 siRNA treatment [42]. The irreversible binding of small cleaved PARP protein is known to prevents DNA repair.

Several anticancer drugs target androgen receptor signaling to prevent tumor growth. Our observation that bergamottin, a natural furanocoumarin found in grapefruit juice, also blocks the androgen receptor expression and activation is very significant as it can be used as a dietary supplement to prevent prostate cancer growth. Previously we have investigated how CYP3A5 inhibitors and inducers can modify AR signaling and effect the efficacy of commonly prescribed concomitant medications [21]. CYP3A4/5 are hepatic enzymes and are involved in processing several commonly prescribed drugs, we do have to be cautious of drug-drug interactions while using bergamottin as an anticancer agent as it is a strong CYP3A inhibitor. Use of bergamottin on the other hand can be very significant for African Americans, who often present aggressive disease, have high AR expression [43, 44], and often carry wild type CYP3A5 [45–47]. Both, the relative incidence and mortality rate are much higher (1.8 and 2.2 times more respectively) in AAs as compared to Caucasian Americans (CA) [16]. Since bergamottin is a natural CYP3A5 inhibitor, combining it with other cancer drugs may provide an opportunity to increase efficacy of treatment,especially in AAs expressing high levels of CYP3A5.

## Conclusions

Bergamottin, a potent CYP3A inhibitor blocks prostate cancer cell growth by inhibiting AR expression, nuclear localization, and PSA production. In addition, it promotes an accumulation of G0/G1 cells causing cell cycle arrest possibly leading to apoptosis and cell death. These observations suggest the potential for bergamottin to be tested as a dietary supplement for prostate cancer prevention and treatment.

## Supporting information

S1 Raw images Full blot of western gels shown in Figs 2, 4 and 5. The dotted lines show places were the blots were cut to treat with separate antibodies. During loading sometime, the markers are not next to the samples, in those cases the representative marker is placed next to the blot.
(PDF)

S1 Fig Uncropped confocal images of Fig 2C and 2D. A z-Stack passing through the middle of the nucleus showing uncropped images corresponding to Fig 2C (LNCaP) and 2D (MDAPCa2b).
(TIF)
