# Supplementary material for: Bergamottin a CYP3A inhibitor found in grapefruit juice inhibits prostate cancer cell growth by downregulating androgen receptor signaling and promoting G0/G1 cell cycle block and apoptosis
Source: PLoS One. 2021 Sep 27;16(9):e0257984. doi: 10.1371/journal.pone.0257984 (PMC8476002; doi:10.1371/journal.pone.0257984)
Supplement: S1 Raw images — The dotted lines show places were the blots were cut to treat with separate antibodies. During loading sometime, the markers are not next to the samples, in those cases the representative marker is placed next to the blot. (PDF) [file pone.0257984.s001.pdf]

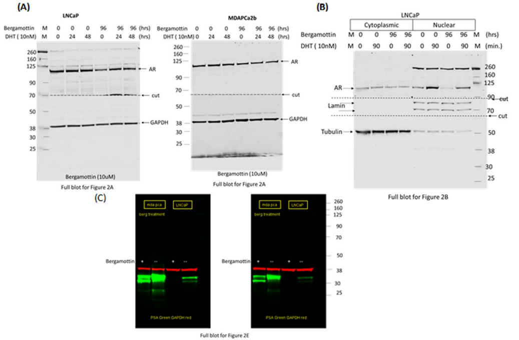

Full blot of western gels shown in Figs 2A, 2B and 2E. The dotted lines show places were blots were cut to treat with seperate antibodies. In (C) the IR dye 700 (red, GAPDH) and IR dye 800 (green,PSA) are shown at two different intensities to show clarity representative of original figure 1E.

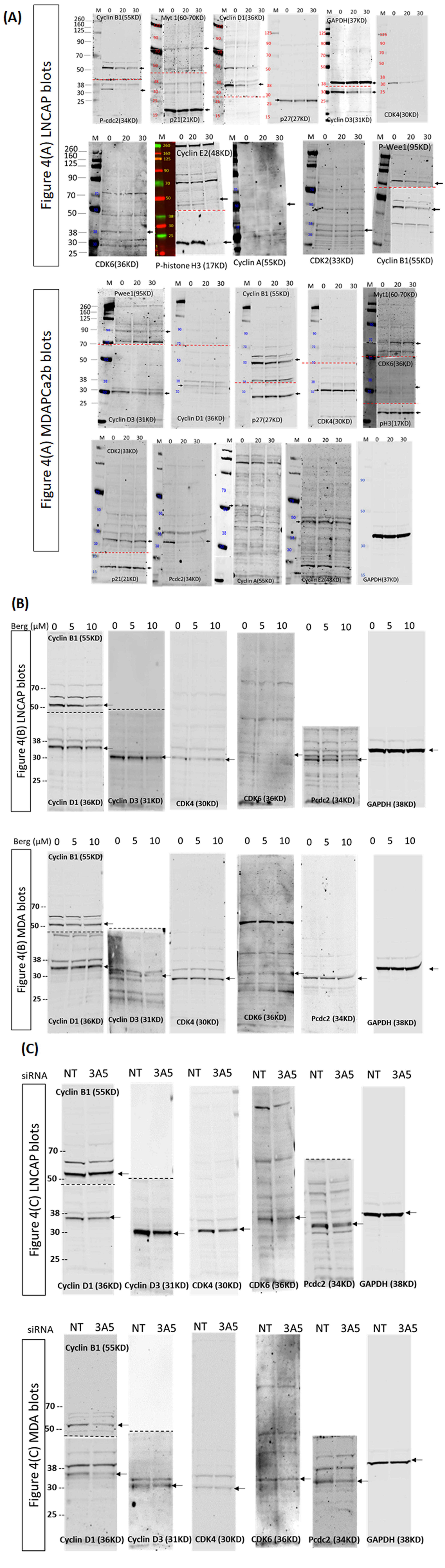

Full blot of western gels shown in Fig 4A, 4B and 4C.
(A)- Blots with LNCaP and MDAPCa2b extracts with high dose bergamottin (20 and 30μM) treatment.
(B)- Blots with LNCaP and MDAPCa2b extracts with low dose bergamottin (5 and 10μM) treatment.
(C)- Blots with LNCaP and MDAPCa2b extracts with NT(non-target) and CYP3A5siRNA treatment.
The dotted lines show places were the blots were cut to treat with separate antibodies. During loading sometime, the markers are not next to the samples, in those cases the representative marker is placed next to the blot.

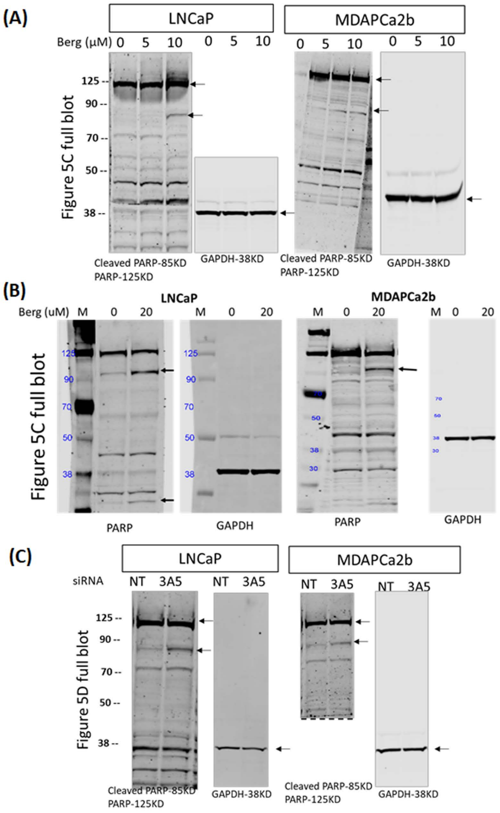

Full blot of western gels shown in Fig 5. (A)- Low dose treatment. (B)- High dose treatment. (C)-Blots with siRNA treatment. Partial blots are where the blot has been cropped for use with other antibodies.
