# Supplementary figures and images for: Bergamottin a CYP3A inhibitor found in grapefruit juice inhibits prostate cancer cell growth by downregulating androgen receptor signaling and promoting G0/G1 cell cycle block and apoptosis
Source: PLoS One. 2021 Sep 27;16(9):e0257984. doi: 10.1371/journal.pone.0257984 (PMC8476002; doi:10.1371/journal.pone.0257984)

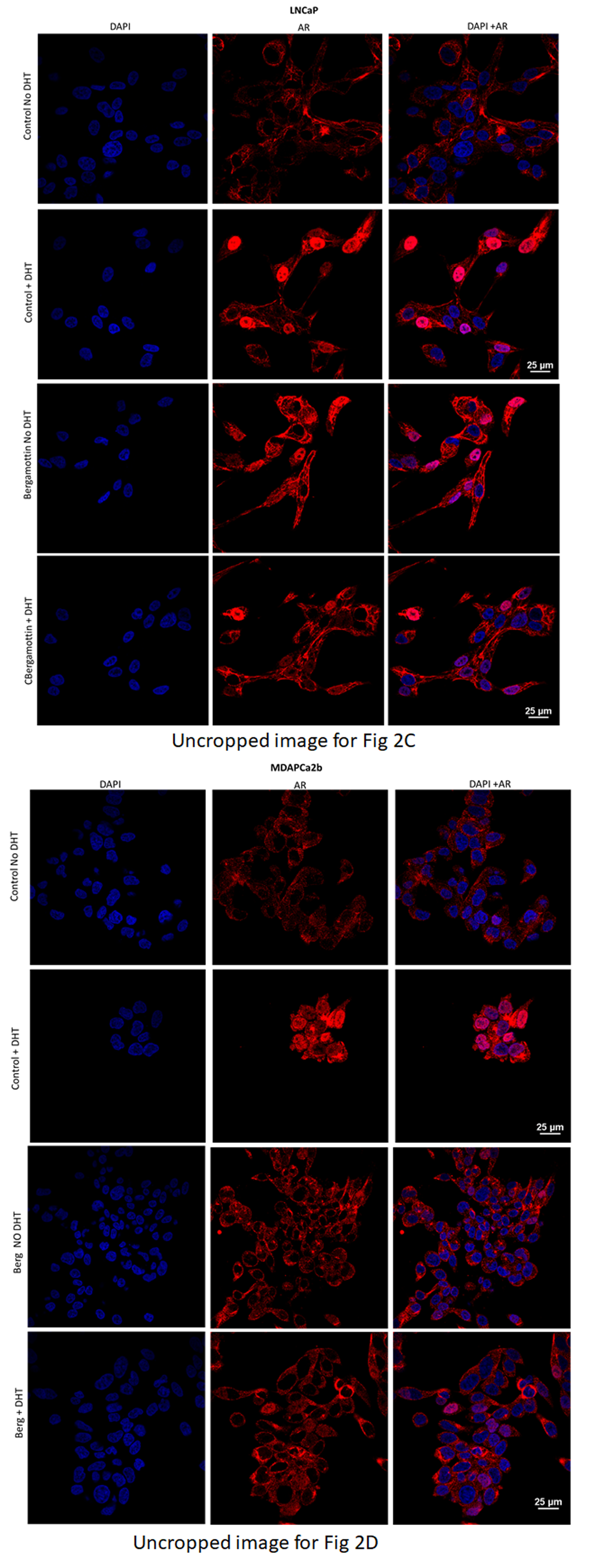

Supplement: S1 Fig — A z-Stack passing through the middle of the nucleus showing uncropped images corresponding to Fig 2C (LNCaP) and 2D (MDAPCa2b). (TIF) [file pone.0257984.s002.tif]
